# Diffusion models for medical image reconstruction

**DOI:** 10.1093/bjrai/ubae013

**Published:** 2024-08-29

**Authors:** George Webber, Andrew J Reader

**Affiliations:** School of Biomedical Engineering and Imaging Sciences, King’s College London, London SE1 7EU, United Kingdom; School of Biomedical Engineering and Imaging Sciences, King’s College London, London SE1 7EU, United Kingdom

**Keywords:** image reconstruction algorithms, diffusion models, score-based generative models, deep learning, MRI, CT, PET, ultrasound

## Abstract

Better algorithms for medical image reconstruction can improve image quality and enable reductions in acquisition time and radiation dose. A prior understanding of the distribution of plausible images is key to realising these benefits. Recently, research into deep-learning image reconstruction has started to look into using unsupervised diffusion models, trained only on high-quality medical images (ie, without needing paired scanner measurement data), for modelling this prior understanding. Image reconstruction algorithms incorporating unsupervised diffusion models have already attained state-of-the-art accuracy for reconstruction tasks ranging from highly accelerated MRI to ultra-sparse-view CT and low-dose PET. Key advantages of diffusion model approach over previous deep learning approaches for reconstruction include state-of-the-art image distribution modelling, improved robustness to domain shift, and principled quantification of reconstruction uncertainty. If hallucination concerns can be alleviated, their key advantages and impressive performance could mean these algorithms are better suited to clinical use than previous deep-learning approaches. In this review, we provide an accessible introduction to image reconstruction and diffusion models, outline guidance for using diffusion-model-based reconstruction methodology, summarise modality-specific challenges, and identify key research themes. We conclude with a discussion of the opportunities and challenges of using diffusion models for medical image reconstruction.

## Introduction

Reconstructing medical images from scanner measurements is a necessary step for many imaging techniques, enabling medical diagnosis and research. Advances in image reconstruction research enable us to accelerate acquisition, improve image quality, or reduce necessary radiation doses.^1^^,^^2^ Sophisticated reconstruction algorithms achieve this by integrating current measurement data, our understanding of imaging physics, and our prior knowledge of plausible reconstructed images.^3^

One recent method for capturing such prior knowledge is the diffusion model,^4^^,^^5^ a state-of-the-art generative deep learning framework for modelling images. Crucially, diffusion models can utilise prior knowledge either with (supervised) or without (unsupervised) knowledge of a specific reconstruction task. By decoupling learning of the prior knowledge from the reconstruction task, diffusion models can overcome existing issues of costly training and poor robustness to varied scan parameters.^6^ As a result, image reconstruction with diffusion models, particularly in the unsupervised setting, is pushing the frontiers of medical image reconstruction.

This review seeks to explain, explore, and review the use of diffusion models for medical image reconstruction. We focus on the inverse problem of image reconstruction, omitting approaches that just perform denoising as a post-processing step.

We begin with an accessible overview of image reconstruction and diffusion model theory, as well as how diffusion models are incorporated into image reconstruction. We offer some practical considerations for working with the algorithms discussed. We then discuss recent advances, including modality-specific research into MRI, CT, PET, and ultrasound imaging. We conclude by considering the challenges and opportunities faced for using these algorithms in a clinical setting.

## Image reconstruction

Image reconstruction is an inverse problem, where we seek an image $\hat{x}$ to explain our noisy scan measurements *m* (eg, sinograms for CT or PET, or k-space data for MRI).^1^^,^^2^ To solve this inverse problem, we first define *A* as the forward model that maps images *x* to their corresponding noise-free measurements *y* = *Ax*. We then find the image estimate with the greatest likelihood L(x;m) of explaining *m*, assuming a model of the noise (ie, L(x;m)=P(m∼Noise(y) | y=Ax)=p(m|x)).

### Incorporating prior knowledge

As measurements become noisier (eg, as scan time or radiotracer dose in PET is reduced) or less complete (eg, to accelerate MRI or CT), the resulting image reconstruction problem becomes highly *ill-posed*, meaning it has no stable, unique solution. We can compensate for this reduction in measurement information by incorporating information about the distribution of probable images *x*, so-called *prior* knowledge. The best-reconstructed image $\hat{x}$ then balances maximising both the likelihood L(x;m), that is, the likelihood that $\hat{x}$ explains the measurements *m*, and the prior *p*(*x*), that is, the probability that $\hat{x}$ is a valid medical image. This image $\hat{x}$ is called the *maximum a posteriori* (MAP) estimate.

### Reconstruction paradigms

As Table 1 summarises, different ways to acquire and use such prior knowledge lead to image reconstruction algorithms with varying strengths and weaknesses.

**Table 1.** A comparison of methods for integrating prior information into image reconstruction.

Method	Training data required	Training difficulty	Reconstruction speed	Reconstruction quality	Resilience to domain shift
Hand-crafted priors	None	N/A (no training required)	Fast	Good	Excellent (no dependency on prior scanner data)
Unsupervised diffusion models	Images	Easy (just training image denoisers)	Slow (complex iterative process with 100+ iterations)	Excellent	Good (at most, weak dependency on scanner)
Supervised learning	Paired measurements and images	Hard (requires learning *A* or calculating gradients through *A*)	Fast (varies, but methods are not inherently slow)	Excellent (best possible with abundant training data)	Poor (strong dependency on training data and its associated scanner(s))

Resilience to domain shift refers to performance on data that is dissimilar to the training data, so-called out-of-distribution data.

Conventionally, using prior knowledge meant minimising hand-crafted functional. For example, we may penalise large variations between neighbouring voxel intensities, resulting in a smoother and less noisy reconstructed image. However, hand-crafted priors are limited by our ability to describe mathematically what complex medical images should look like.^2^

Viewing reconstruction as a supervised deep learning problem offers a data-driven way to incorporate more complex prior information, by learning a prior from measurements and corresponding high-quality images.^1^ For example, we may train a neural network to learn a mapping from low-quality measurement data to high-quality images, resulting in a network that can perform reconstruction tasks.^7^ However, the large volumes of paired training data required are difficult to acquire. Furthermore, supervised training results in reconstruction algorithms that are not robust to domain shift, performing poorly on data that is different to their training data (eg, data acquired with different scanning parameters).

An alternative paradigm is unsupervised reconstruction, which uses an image prior to learning without knowledge of the reconstruction task.^1^ For example, we may incorporate an untrained deep image prior (DIP) or generative image model to regularise our reconstruction process.^8^ As measurement data is not needed for training, neither is the forward model *A*; this helps to decouple the learnt prior from particular scanning parameters. This can lead to faster training, lower training data requirements, and greater robustness to domain shift than supervised reconstruction methods. However, the learnt prior is necessarily less specific to the reconstruction task than in supervised reconstruction, so it risks sacrificing accuracy for flexibility.

## Why diffusion models?

### Comparison of diffusion models to other generative models

Diffusion models belong to the machine learning paradigm of deep generative models. Generative models use training images to learn a prior probability distribution of images (eg, brain PET or cardiac MRI images), from which they then sample to generate new images.

In recent years, *diffusion models*^4^^,^^5^ have become the state-of-the-art generative model for learning distributions of images. Compared to autoencoder and normalising flow approaches, diffusion models generate higher fidelity samples.^9^ Compared to adversarial learning approaches such as generative adversarial networks (GANs), they offer superior mode coverage, that is, samples match the diversity of the relevant distribution of medical images more closely.^9^^,^^10^ While diffusion models typically take longer to generate images than other popular approaches, this is usually an acceptable trade-off for higher-quality image reconstruction. See Table 2 for a more detailed comparison.

**Table 2.** Comparison of 3 popular generative image modelling frameworks.

Method	Training stability	Image quality	Generation time	Mode coverage
Autoencoder (eg, VAEs, WAEs)	Good (regularised reconstruction objective is usually simple to balance)	Medium (typically lower than state-of-the-art)	Fast (varies with methods, but typically faster than diffusion models)	Good (variational inference techniques help approximate the whole distribution)
Adversarial learning (eg, GANs)	Poor (difficulty balancing the competing actors in adversarial training)	Good (state-of-the-art)	Fast (varies with methods, but typically faster than diffusion models)	Poor (adversarial training objectives are prone to mode collapse)
Diffusion model (eg, DDIM, DDPM)	Good (just requires training denoisers at multiple noise scales)	Good (state-of-the-art)	Slow (computationally heavy iterative process)	Good (inherent randomness helps to generate diverse samples)

Abbreviations: DDIM = denoising diffusion implicit model, DDPM = denoising diffusion probabilistic model, GAN = generative adversarial network, VAE = variational autoencoder, WAE = Wasserstein autoencoder.

Diffusion models are therefore a natural choice of deep learning framework to integrate with inverse problem solving and more specifically medical image reconstruction.

### Comparison of diffusion model reconstruction to state-of-the-art reconstruction

Diffusion models are inherently iterative and may be integrated with existing model-based iterative methodology to yield unsupervised reconstruction algorithms that leverage the image modelling abilities of diffusion models. As previously discussed in Reconstruction paradigms, such algorithms benefit from lower training data requirements and greater robustness to domain shift than supervised reconstruction methods. In particular, the same trained model may be used at differing acceleration factors or coil configurations for MRI, or dose levels for PET.

Furthermore, once a diffusion model has been trained and conditioned on measured data, it implicitly models the full posterior distribution of possible reconstructed images (whereas conventional methods just provide a point estimate such as the MAP estimate). In particular, by generating multiple plausible reconstructions, we may quantify reconstruction uncertainty at the voxel level.

In summary, the potential advantages of using unsupervised diffusion models for medical image reconstruction include:

- State-of-the-art image generation, with superior image quality and diversity to other generative modelling methodologies.
- Improved training and handling of domain shift, as a result of decoupling the image prior from the scanner parameters.
- The ability to sample from the posterior distribution of possible reconstructions, and thereby quantify reconstruction uncertainty.
- Natural integrations with existing model-based iterative reconstruction algorithms.

However, in use cases with many high-quality measurement datasets and unchanging scanner parameters, state-of-the-art supervised reconstruction retains an advantage over unsupervised diffusion due to the additional information in measurement data.

## Diffusion model theory

### Diffusion models as a noising and denoising process

**Figure 1.**
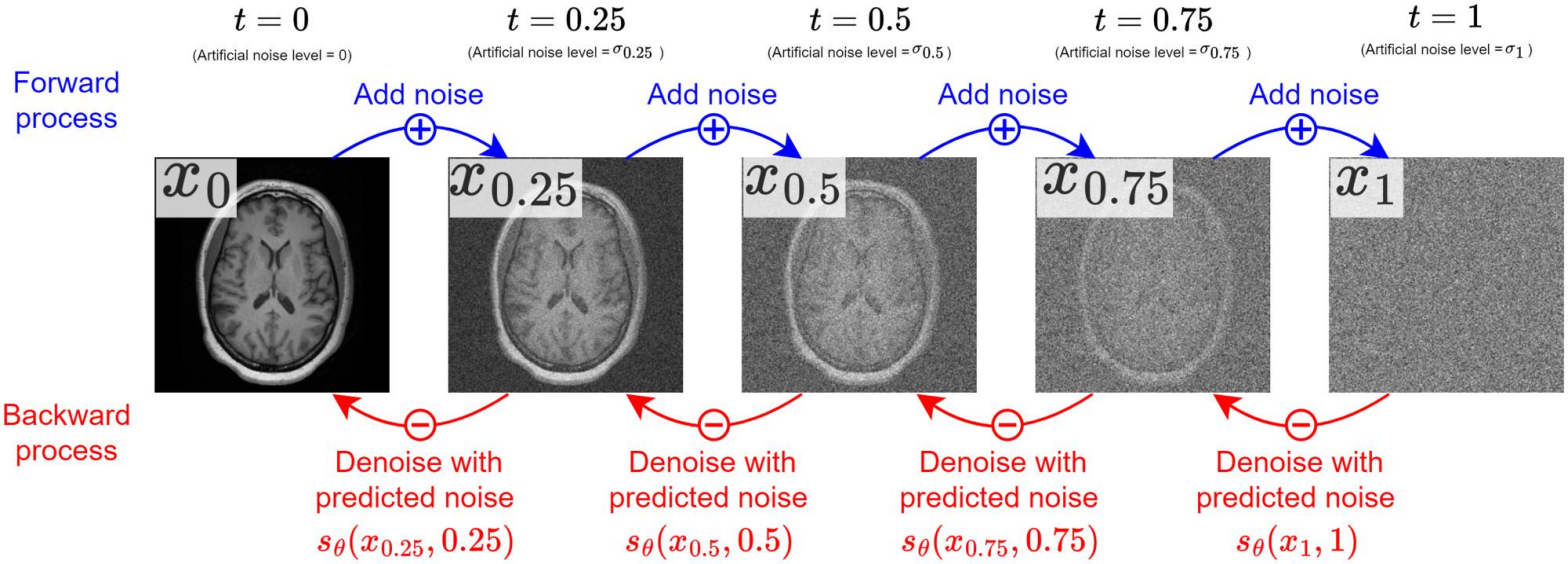
A diffusion model’s iterative noising (additive) and denoising (subtractive) process, shown for *T* = 4 timesteps with a single example image.

Diffusion models consist of 2 iterative processes, both shown in Figure 1: a random *forward* process and a generative *backward* process.

The forward process is simple: we take an image *x*_0_ as input and corrupt it repeatedly by adding artificial random Gaussian noise for *T* steps (indexed by time $t=0,\frac{1}{T},\frac{2}{T},\ldots ,1$). This process yields a pure noise image *x*_1_ where all original information has been lost.

The backward process is the reverse of the forward process. We generate a pure noise image *x*_1_ and iteratively denoise it for a fixed number of timesteps (eg, yielding a sequence of less-noisy images $x_{0.99},x_{0.98},x_{0.97},\ldots ,x_{t},\ldots$). After *T* timesteps, the output *x*_0_ is a high-quality image belonging to the same distribution of images as the training data.

**Figure 2.**
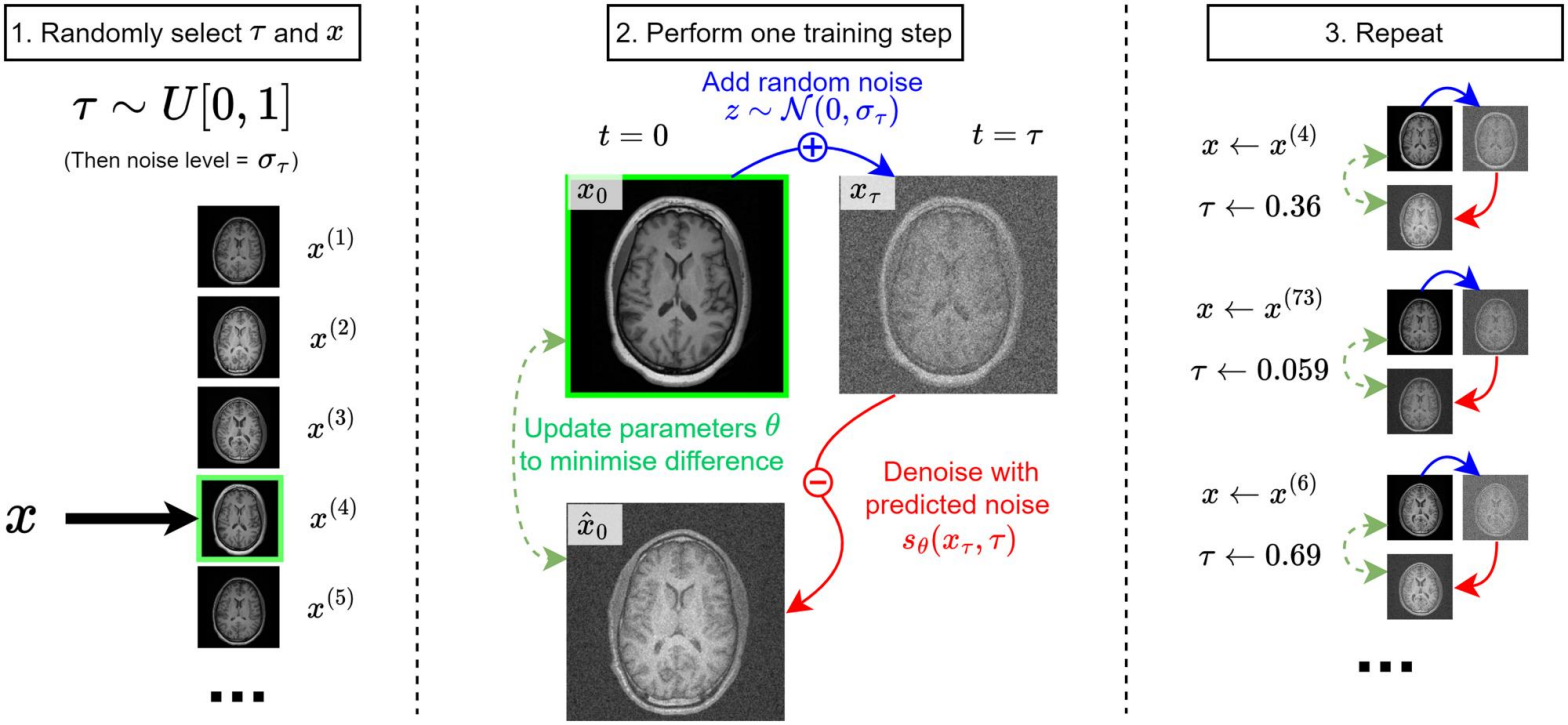
One possible training algorithm for a score-based generative model (SGM) with noise-level-dependent network $s_{\theta }$. Using this training algorithm $s_{\theta }$ is trained to map artificially noisy versions of training images (with any randomly selected level of artificial noise) to their denoised counterparts. Alternative formulations learn instead to predict $x_{\tau }$ from $x_{\tau +\epsilon }$, or select *τ* from a predefined discretisation of [0,1]. Side information can be incorporated by conditioning $s_{\theta }$, for example, providing a guidance MRI image for a diffusion model trained on PET images.

The denoising step is learnt by training a neural network to remove different levels of artificial noise from images, a process shown in Figure 2. This is typically done with a single noise-level-dependent neural network $s_{\theta }(x_{t}\;|\;t)$, which is given the current timestep *t* and image *x_t_* as inputs.

Once the denoising step has been learnt, a diffusion model can generate realistic medical images from noise. The iterative denoising can be implemented by directly predicting $x_{t-1}$ from *x_t_* using $s_{\theta }$, in the case of DDPMs (denoising diffusion probabilistic models).^5^ An important modification to DDPMs is DDIMs (denoising diffusion implicit models).^11^ A DDIM uses the same neural network $s_{\theta }$ as a DDPM but removes noise from *x_t_* by estimating the fully denoised image *x*_0_ and adding back noise to get $x_{t-\epsilon }$; this can lead to 10–50× faster image generation by skipping timesteps.

### Diffusion models as score-based generative models

**Figure 3.**
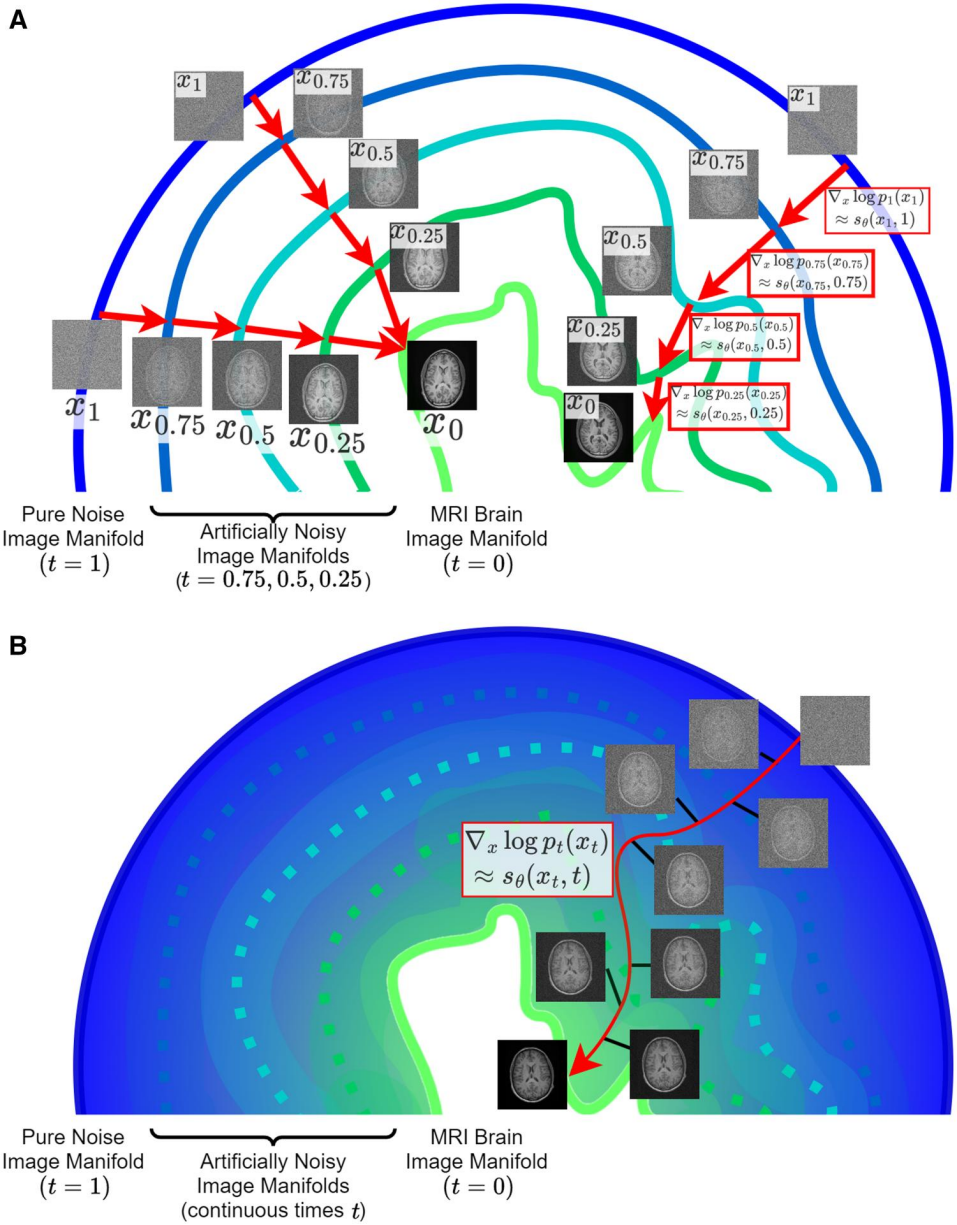
A 2D visualisation of score-based generative models (SGMs) from the perspective of artificially noisy image manifolds. (**A**) The coloured line segments each represent a manifold containing all possible artificially-noised medical images from our distribution of interest, at a given discrete noise level *σ_t_*. The 9 brain images on the left visualise the diffusion model process of converting noise images into true medical images (note that this mapping may not be a one-to-one function). The path on the right shows the same process, with additional labels showing how the score vector is approximated by $s_{\theta }$ to guide image generation towards the medical image manifold without artificial noise. (**B**) Discrete noise levels are replaced by a continuous (monotone) noise schedule; in this setting, we see how the score function charts a continuous path from a pure noise distribution to the desired image manifold. We may discretise this process into arbitrarily small timesteps to generate high-quality images.

In the image reconstruction literature, diffusion models are more often formulated from the score-based generative model (SGM) perspective^12^ (see Figure 3). The central concept in the SGM formulation is the score $\nabla_{x}\;\text{log}\;p(x)$, a vector that points from image *x* towards highly probable images.

The score can hence guide a generative process, but generating images with the score alone is computationally infeasible for medical images.

To make this process tractable, we learn approximate versions of the score, by learning to calculate the score for noisy images at decreasing noise levels $\sigma_{1}>\ldots >\sigma_{\frac{2}{T}}>\sigma_{\frac{1}{T}}>0$. Then, to generate samples, we start with pure noise and use decreasingly noisy score vector approximations to guide an iterative algorithm towards a highly probable image. Figure 3A visualises how score functions learnt on artificially noisy data guide the generation of high-quality images.

**Figure 4.**
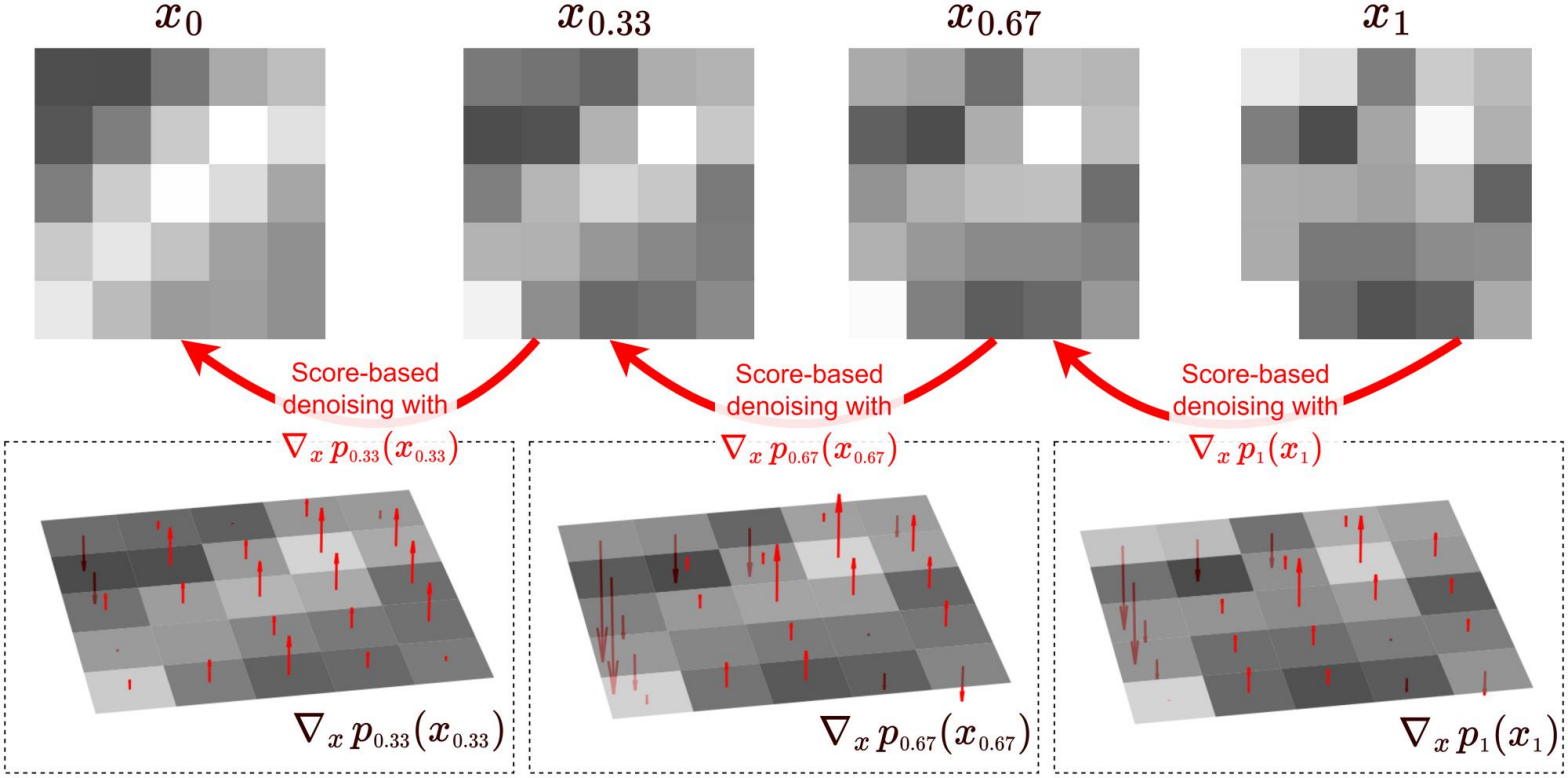
Diffusion model image generation from the perspective of the score vector $\nabla_{x}p_{t}(x_{t})$. Here we show *T* = 3 equal timesteps of the backwards diffusion process for a small 5 × 5 patch, and for each timestep, we visualise the score vector as an array of arrows in red. It may be observed (particularly from the top left pixels) that the score function at each timestep points in the direction that reduces the noise in the image (where an “up” arrow represents an increase in a pixel value, ie, lighter, and a “down” arrow represents a decrease in a pixel value, ie, darker).

We may learn the approximate score vectors $\nabla_{x}\;\text{log}\;p_{t}(x_{t})$ with a noise-level-dependent neural network $s_{\theta }(x\;|\;t)$ (using the Denoising Score Matching algorithm), see Figure 4 for a visualisation.

From the above description, it is hopefully not surprising that our previous formulation for “denoising” is broadly equivalent to the SGM formulation.^13^ We may therefore view training and sampling from diffusion models in terms of learnt denoising or guiding image generation with the score function. The SGM perspective, while more mathematically involved, has the advantage of showing how to model the probability distribution *p*(*x*) of images explicitly.

### Diffusion models as continuous random processes

The natural generalisation of the SGM framework is to consider the SGM as a discrete version of a continuous noising process (see Figure 3B). This leads to the stochastic differential equation (SDE) formulation of SGMs.^14^

In practice, this allows us to forgo specifying a fixed set of *T* noise levels in training, instead of specifying a continuous noise schedule. When we train our model we can then sample random noise levels from the schedule. This is beneficial for image generation, as it allows us to vary the number of timesteps used (and hence the image quality/speed of generation) without retraining the model.

## Integrating diffusion models with image reconstruction

### Unsupervised reconstruction

**Figure 5.**
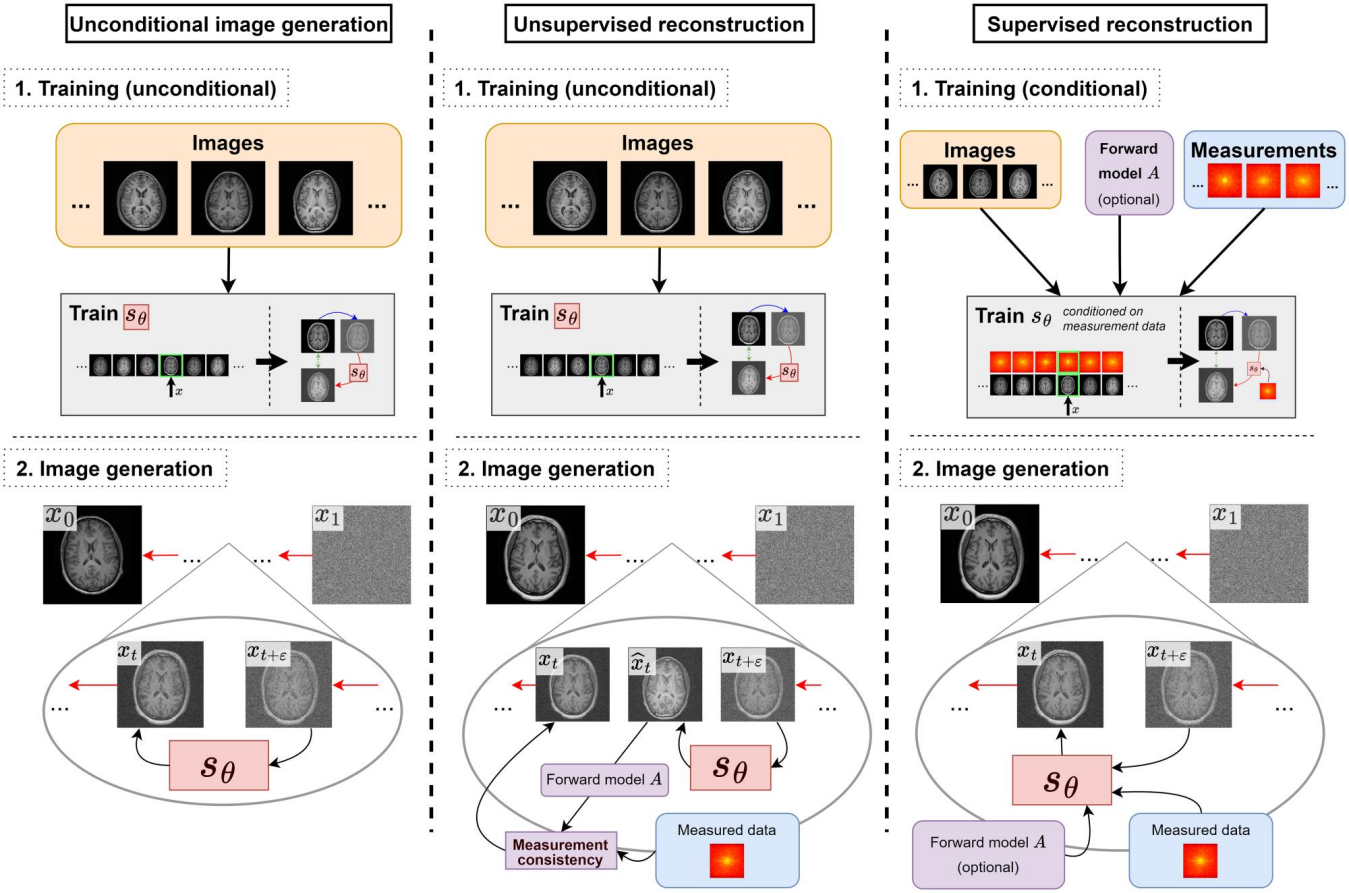
Varying paradigms for using diffusion models for image reconstruction; this review focuses on unsupervised reconstruction. Left: To generate images from the training distribution, the score network $s_{\theta }$ is first trained on example images (see Figure 2). Then, to generate a new image, pure noise *x*_1_ is given as input, and iteratively denoised using $s_{\theta }$ until a new high-quality sample *x*_0_ remains. Middle: To reconstruct in the absence of paired training data, unsupervised reconstruction can be performed. As in the leftmost panel, the score network $s_{\theta }$ is first trained on example images. Then, to perform reconstruction, the iterative denoising process is interleaved with steps to promote consistency with measured data. Right: If we have paired training data, we may condition our score network $s_{\theta }$ on the measurement data. Optionally, the forward model *A* can be incorporated into this process; otherwise, the scanner physics must be learnt by the model. Training in this case learns a conditional score network $s_{\theta }$. To perform reconstruction, the learnt network is conditioned on the measured data and used directly to generate a reconstructed image *x*_0_ from a pure noise image *x*_1_. (This case is representative of conditional generation.)

The prior distribution of images learnt by a diffusion model may be exploited to solve inverse problems in medical imaging.^6^^,^^15^^,^^16^ Most simply, this is achieved by interleaving the diffusion model’s generative denoising steps with additional steps to encourage consistency with measured data (see Figure 5).

More formally, at timestep *t*, instead of the noisy score $\nabla_{x}\;\text{log}\;p_{t}(x_{t})$, we use (an approximation of) the *conditional* noisy score $\nabla_{x}\;\text{log}\;p_{t}(x_{t}|m)$ to guide our image generation process. We can decompose this term using Bayes’ Law:

$$\nabla_{x}\;\text{log}\;p_{t}(x_{t}|m)=\nabla_{x}\;\text{log}\;p_{t}(x_{t})+\nabla_{x}\;\text{log}\;p_{t}(m|x_{t})$$

The first term $\nabla_{x}\;\text{log}\;p_{t}(x_{t})$ is just the unconditional noisy score so can be learnt as we’ve seen already. However, the second term $\nabla_{x}\;\text{log}\;p_{t}(m|x_{t})$, that is, the noisy score of the likelihood of the noisy image, is difficult to calculate. This is because for t≠0 there is a mismatch between our likelihood function *L* for noise-free (ie, *t* = 0) data and the noisy iterate *x_t_* we would like to apply it to.

The likelihood term $\nabla_{x}\;\text{log}\;p_{t}(m|x_{t})$ can be approximated by naïvely scaling the usual log-likelihood gradient $\nabla_{x}\;\text{log}\;p(m|x)$, which is fast but inaccurate.^15^^,^^17^ Diffusion posterior sampling (DPS)^18^ instead proposes calculating the gradient of a combined “denoise-and-apply-likelihood” operation, which is slow but highly accurate. Other approaches such as decomposed diffusion sampling (DDS)^19^ forgo direct approximation and instead apply the likelihood to the estimate of *x*_0_ in the DDIM generation process.^20^

The choice of approximation method depends on the speed of reconstruction required, the size of the image output, the noise in the image, and the particular forward model in use.

### Supervised reconstruction

While not the focus of this article, diffusion models may be used for familiar supervised learning, by inputting the measurement data to $s_{\theta }$ in the training process. Of course, such an approach no longer has the aforementioned benefits of alleviating the key issues of training data requirements and domain shift. Figure 5 illustrates the key paradigms for using diffusion models for reconstruction.

## Practicalities

For $s_{\theta }$, most researchers use a U-Net architecture with at least 3 down and upsampling stages with^6^ or without^14^^,^^17^^,^^21^ attention components, with a sinusoidal embedding for the timestep *t*. Some authors instead use variants of the vision transformer architecture.^22–24^ Most papers report training for 100-50 000 epochs (with most < 5000).^6^^,^^24–29^ Ideally, training should use as many images as possible and should continue until the loss on a validation set has been minimised (assessed with fixed timesteps and fixed random seed for stability). Adam with learning rate $∼10^{-4}$ is a common choice of optimiser.^30–32^ Image generation (inference) is typically performed for 100-4000 iterations,^21^^,^^33^ depending on image dimensions and noise schedule choice. Longer inference usually results in better samples, with diminishing returns expected beyond 1000 iterations.

Simple noise schedule choices include linear or cosine schedules.^34^ The final variance should be set such that noisy images at this noise level approximate pure Gaussian noise (this should be checked visually and statistically).

Images should be cropped to remove empty space, and normalised before input for improved training. See the section on 3D reconstruction for handling of 3D data.

Most authors implement their work in Python, using either PyTorch (more flexible) or TensorFlow (shallower learning curve). Open-sourced code is an extremely valuable starting point; see Chung and Ye^6^ (CT/MRI) or Singh et al^21^ (PET), for example, implementations, and Algorithms 1 and 2 for pseudocode.

Algorithm 1 Training a score-based generative model

**Input:** Untrained neural network $s_{\theta }$ with parameters *θ*

**Input:** Noising process ν(t)

**Input:** Image data $x^{\left(1\right)},x^{\left(2\right)},\ldots ,x^{\left(M\right)}$

**Input:** Epoch number *N*

**Output:** Trained neural network $s_{\theta }$

 Initialise optimiser *opt*

 **for**i=1,2,…,N**do** ▹ Epoch for loop

  Shuffle images $x^{\left(1\right)},x^{\left(2\right)},\ldots ,x^{\left(M\right)}$

  **for**j=1,2,…,M**do** ▹ Image for loop

   t∼U[0,1] ▹ Get random timestep

   z∼N(0,I) ▹ Sample noise

   $\mu_{t},\sigma_{t}\leftarrow \nu (t)$ ▹ Get noising parameters

   $x_{t}^{\left(j\right)}\leftarrow \mu_{t}\cdot x^{\left(j\right)}+\sigma_{t}\cdot z$ ▹ Define noisy image

   $\hat{\epsilon }\leftarrow s_{\theta }(x_{t}^{\left(j\right)},t)$ ▹ Predict score (negative noise)

   L$\leftarrow ||\hat{\epsilon }+z|{|}_{2}^{2}$ ▹ Calculate loss

   θ←*opt*($s_{\theta },\;L$) ▹ Improve *θ* by minimising L

  **end for**

 **end for**

 **return**$s_{\theta }$

Algorithm 2 Image generation (or reconstruction) with a pre-trained score-based generative model

**Input:** Trained neural network $s_{\theta }$

**Input:** Time discretisation $0=t_{k_{1}}\leq \ldots \leq t_{k_{T}}=1$

**Input:** Noising process ν(t)

 **Optional:** measurement data **y**, forward model *A*

**Output:** Generated sample **x**

 $x_{1}∼N(0,I)$ ▹ Get initial noisy image

 **for**k=T−1,T−2,…,1**do**

  ${\hat{\epsilon }}_{t_{k+1}}\leftarrow s_{\theta }(x_{t_{k+1}},t_{k+1})$ ▹ Estimate score

  $\mu_{t},\sigma_{t}\leftarrow \nu (t)$ ▹ Get noising parameters

  $x_{t_{k}}\leftarrow \text{REMOVE}-\text{NOISE}({\hat{\epsilon }}_{t_{k+1}},\mu_{t},\sigma_{t})$

  **if**y,A provided **then**

   $x_{t_{k}}\leftarrow \text{CONDITION}-\text{ON}-\text{MEASUREMENTS}(x_{t_{k}},y,A)$

  **end if**

 **end for**

 **return**$x_{0}$

## Modality-specific challenges

Different modalities in medical imaging come with their own challenges. For example, PET data is usually fully sampled with high-variance Poisson noise, while MRI data is usually undersampled with lower-variance Gaussian noise. As a result, the reconstruction problems associated with each modality are related but different.

In addition, modality-specific use cases inform different research focuses (eg, motion correction for cardiac MRI^35^) In this section, we survey some of the attempts to solve modality-specific challenges to image reconstruction with diffusion model methodology.

### Magnetic resonance imaging

A major focus for MRI reconstruction is accelerating acquisition. Much successful work combines modern diffusion models with classical techniques for MRI acceleration such as compressed sensing,^15^^,^^36^ parallel imaging,^28^^,^^37–41^ and the use of non-Cartesian sampling trajectories.^42^ The resulting algorithms generally report improved image quality at higher acceleration factors, and improved robustness to domain shift.

Many sub-problems for MRI reconstruction have also been addressed, for example, motion correction for high-resolution foetal^43^ and adult^35^^,^^44^ brains. Safari et al^35^ note that these methods are particularly beneficial for dealing with diverse involuntary motions in elderly patients.

Other avenues explored include utilising multi-contrast MRI information,^45^^,^^46^ dynamic MRI,^47–49^ quantitative MRI,^27^ and generating high-field images from low-field data.^29^

#### MRI case study

**Figure 6.**
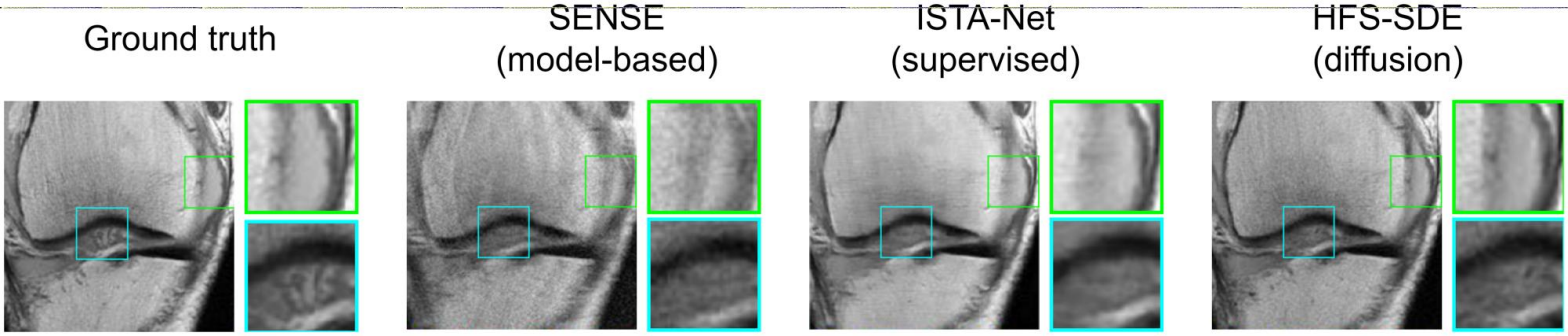
Example diffusion-based reconstruction of 12-fold undersampled knee MRI, using the HFS-SDE (HFS = high-frequency space) methodology proposed by Cao et al.^50^ The conventional SENSE^51^ parallel imaging method (column 2) yields a reconstruction with artefacts. In contrast, the deep learning methods (columns 3 and 4) yield higher-quality reconstructions, with HFS-SDE (column 4) achieving higher reconstruction accuracy than the supervised deep learning method (column 3). Figure courtesy of Yanjie Zhu.^50^ SDE = stochastic differential equation.

Cao et al^50^ propose HFS-SDE (HFS = high-frequency space), a diffusion-based approach tailored to MRI that is focused on reconstructing high-frequency image details from undersampled data—see example results in Figure 6. On ∼6× accelerated data, their algorithm can decrease the reconstruction error by 10× relative to conventional parallel imaging reconstruction (from 14% to 1.4%) and performs competitively with supervised deep learning methods that require retraining for each different undersampling mask.

### Computed tomography

Most diffusion-model-based reconstruction work on CT is for sparse-view^33^^,^^52–58^ or limited-angle CT,^57^^,^^59–61^ where the learnt prior is used to compensate for incomplete data.

Low-dose CT has also been addressed,^16^^,^^62–64^ where the learnt prior compensates for a worse signal-to-noise ratio. A key distinction between efforts is whether the diffusion model acts on image space only (eg, Chung et al^19^), projection space only (eg, Guan et al^54^), or both synergistically (eg, Pan et al,^55^ Xia et al^56^ or Li et al^65^).

Some CT geometries are inherently 3D, so are especially exposed to challenges with 3D reconstruction^55^^,^^56^^,^^66^ (see 3D reconstruction). Additionally, spectral CT has been successfully integrated with diffusion reconstructions,^67–69^ while Zhou et al^70^ have considered the non-standard geometry of robotic CT. In both of these cases, authors found improved image resolution relative to standard model-based iterative reconstruction algorithms.

#### CT case study

**Figure 7.**
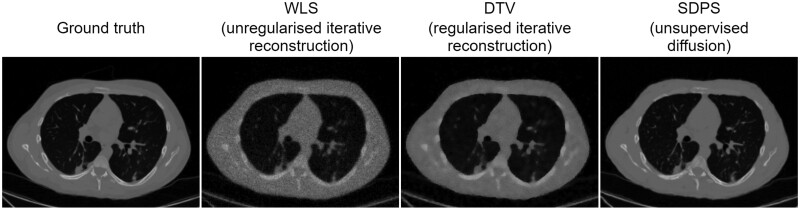
Reconstruction results of a multiple energy bin chest CT scan (80 keV energy bin shown) with fan beam geometry and 120 angles. Column 1: ground truth reference image. Column 2: unregularised iterative reconstruction with weighted least-squares (WLS). Column 3: state-of-the-art synergistic reconstruction with directional total variation prior (DTV). Column 4: unsupervised diffusion model adapted to a synergistic reconstruction setting, using Vazia et al’s^68^ spectral diffusion posterior sampling (SDPS) method. Figure courtesy of Corentin Vazia.^68^

Vazia et al^68^ propose spectral diffusion posterior sampling (SDPS), an adaptation of unsupervised diffusion reconstruction to tackle synergistic reconstruction for spectral CT. They show empirically on real data that their method is efficient, robust to different levels of noise, and outperforms state-of-the-art iterative regularised reconstruction. Figure 7 shows example reconstructions for the 80 keV energy bin. For the example shown, SDPS achieves a structural similarity index measure of 0.87, compared to 0.79 for the previous state-of-the-art and just 0.49 for the unregularised method.

### Positron emission tomography

Variations in injected dose and differences between patients can lead to wide differences in dynamic range between PET images, potentially hindering the training of a diffusion model. Singh et al^21^ address this with a novel normalisation scheme for images before training and during reconstruction. Noise-level-aware diffusion models are an unexplored alternative seen in a denoising context, as done by Xie et al.^71^

Another PET-specific challenge is that the Poisson noise model introduces non-negativity constraints when calculating the likelihood L(x;m). Existing work has clamped negative values to be zero in the likelihood calculation,^21^ although this risks inadequately guiding the reconstruction at early iterations when noise is still dominant; replacing the usual Gaussian noising process with a non-negative noising process has been suggested.^21^

Other avenues of exploration include MRI-guided PET,^21^ joint PET-MRI reconstruction,^72^ and ultra-low-dose PET reconstruction.^73^^,^^74^

#### PET case study

**Figure 8.**
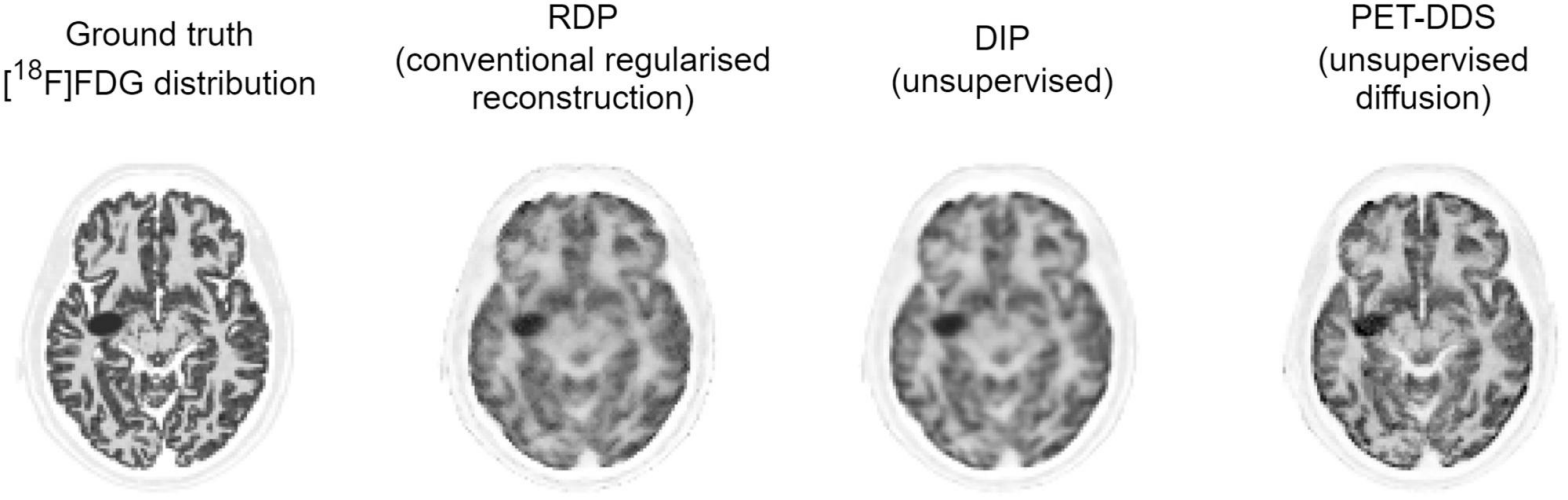
Example diffusion-model-based reconstruction of high-quality simulated [^18^F]FDG-PET brain scans with an artificial lesion. Column 1: ground truth image. Column 2: reconstruction with a conventional maximum likelihood algorithm with relative difference prior (RDP) regularisation. Column 3: reconstruction with the unsupervised deep image prior (DIP) algorithm. Column 4: reconstruction with Singh et al’s^21^ unsupervised diffusion method PET-DDS. Figure courtesy of Imraj Singh.^21^

Singh et al^21^ propose memory-efficient methods for full 3D PET reconstruction. They show comparable image quality to existing state-of-the-art supervised methods while showing improved contrast recovery coefficient values for simulated lesions (eg, 98% vs 87% for supervised methods). Representative reconstructions versus other unsupervised methods are shown in Figure 8.

#### Disambiguation

A common misconception is that PET’s Poisson noise model is incompatible with the artificial Gaussian noise used in the diffusion process. This is not an issue. In general, the diffusion process maps between a useful distribution of medical images (with some inherent noise) and a known distribution. This known distribution is chosen to be Gaussian for its nice mathematical properties, though other options are possible. This Gaussian choice of known distribution is separate from the *inherent* Gaussian noise in some medical image modalities (eg, MRI, CT).

### Ultrasound

Ultrasound imaging faces the challenge of poor signal-to-noise ratio, as well as highly structured and correlated noise.

Some unsupervised diffusion model approaches (eg, Lan et al,^75^ Zhang et al^76^ and Merino et al^77^) have reported significant improvements over the conventional delay-and-sum reconstruction technique and comparable results to the state-of-the-art for plane wave ultrasound imaging.

In the radiofrequency domain, ultrasound data can have a high dynamic range, making it difficult to learn priors. Stevens et al^78^ explore solutions to this problem for the use case of dehazing cardiac ultrasound images.

#### Ultrasound case study

**Figure 9.**
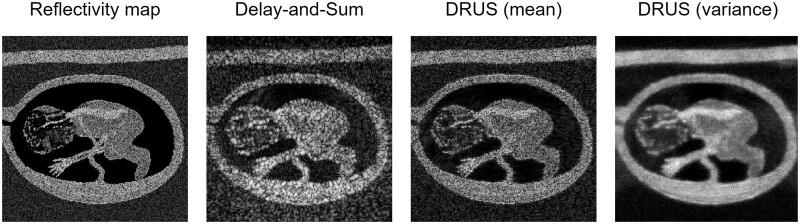
Example diffusion-model-based reconstruction of a simulated foetal ultrasound scan, using Zhang et al’s^79^ DRUS (diffusion reconstruction of ultrasound images) method. The diffusion-based reconstruction (column 3) shows improved image resolution and similarity to the true echogenicity map (column 1) compared to the standard delay-and-sum technique (with 1 plane wave transmission) (column 2). The variance of the reconstruction is also computed (column 4), highlighting increased uncertainty at tissue boundaries. Figure courtesy of Yuxin Zhang.^79^

Zhang et al^79^ show sampling multiple possible reconstructions with a diffusion model and computing the variance image to be helpful for despeckling images, as Figure 9 shows. On phantom datasets, this technique shows an improved contrast-to-noise ratio against the gold standard (18.4 dB vs 6.7 dB delay-and-sum with 75 plane wave transmissions) but does not improve axial resolution as much as supervised reconstruction techniques (eg, 0.29 mm full-width half-maximum resolution vs 0.22 mm for supervised vs 0.38 mm for delay-and-sum).

### Other modalities

To date, diffusion model reconstruction has also been explored in photoacoustic tomography,^80^^,^^81^ electrical impedance tomography,^82^ and electroencephalography,^83^ where it has generally shown greater reductions in noise and improved generalisation to new measurement processes than conventional reconstruction techniques.

## Key research directions

Image reconstruction with diffusion models is a highly active research field; last year alone saw an approximately 3-fold year-on-year increase in publications. In this section, we synthesise the field’s key research directions, with a focus on unsupervised diffusion models.

### Resolving mismatches between prior and measurements (avoiding hallucinations)

**Figure 10.**
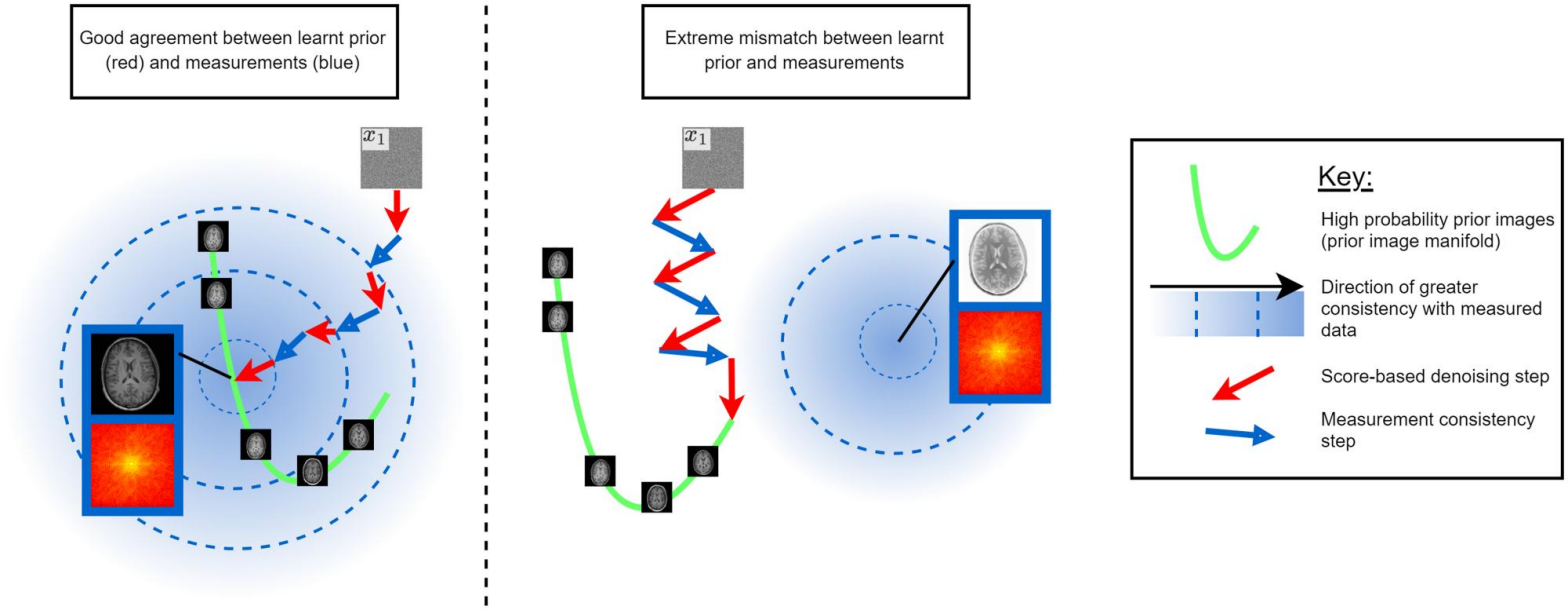
Visualisations of unsupervised diffusion model reconstruction, in cases of good (left) and poor (right) agreement between the prior and measurement likelihood. A diffusion model trained on irrelevant or poor-quality data can hinder rather than help reconstruction, or worse, lead to high-quality reconstructions inconsistent with reality (a so-called hallucination). Resolving small mismatches between the measurement consistency steps and the score-based denoising steps (due to noise or differences between training images and images to be reconstructed) is a subject of research interest.

Often, training images have systematic differences from the image we seek to reconstruct,^84^ for example, if training images are from a different scanner or hospital. In this case, steps to promote measurement consistency can hinder agreement with the diffusion prior and vice-versa (see Figure 10 for an extreme example). This can lead to hallucinations, where a high-quality reconstruction that is inconsistent with reality is produced.^85^

Some approaches attempt to resolve the mismatch via better measurement-conditioning schemes.^18^^,^^53^^,^^86^ Chung et al^18^ proposed resolving the mismatch by projecting the likelihood gradient onto the manifold of the diffusion prior, but this does not account for the noise in measurement data. DPS^86^ is an improvement in this regard but is slow as it requires backpropagation through the score network $s_{\theta }$.

Other approaches attempt to adapt the diffusion prior to the current reconstruction task via self-supervision. Güngör et al^87^ fine-tune deep learning parameters controlling the prior to minimise measurement-consistency loss, outperforming other methods on out-of-distribution data. However, this approach is specific to their reconstruction architecture. Barbano et al^88^ propose steerable conditional diffusion (SCD), a more general learning-free approach based on low-rank adaption (LoRA)^89^ that drastically reduces hallucinatory features on out-of-distribution data. Chung and Ye^90^ recently extended this work via a connection with the DIP, proposing a more efficient adaptation method for 3D inverse problem-solving in challenging imaging applications. This line of work is promising, not least due to the success of fine-tuning in the large language model field.^89^

### Accelerating reconstruction times

Diffusion models have slow image generation, often taking thousands of iterations to generate high-quality images.^39^ This is a key barrier to clinical use. Some efforts have integrated advances in diffusion model theory (eg, DDIMs) to yield faster image reconstruction algorithms.^21^^,^^26^ Particularly of interest are Zhao et al^23^ and Güngör et al’s^87^ use of adversarial training to enable generation with fewer timesteps, due to the success of GANs for supervised image reconstruction.

Other approaches are specific to image reconstruction, where we have the advantage of easily obtaining a cheap image estimate. Some authors have attempted “hot starts”, that is, running the diffusion process starting from a conventional image estimate.^26^ However, this estimate is unlikely to lie on an artificially noisy image manifold where the diffusion model was trained. Chung et al^91^ suggest a neural network adaptation to overcome this, claiming stable state-of-the-art results on knee MRI with 10–50× fewer iterations. A slower, but more widely explored, alternative is “coarse-to-fine sampling”, where a coarse estimate is first generated from the diffusion model, followed by a second process of fine denoising.^32^^,^^87^^,^^92^

A separate approach has been to integrate reconstruction with acceleration schemes for existing iterative reconstruction algorithms (eg, Nesterov momentum, alternating direction method of multipliers algorithm).^39^^,^^63^

### Learning from imperfect training data

It can be impossible to obtain high-quality training images ideal for training a diffusion model. For example, cardiac MRI acquisitions are undersampled to fit within a breath hold and CT acquisitions have limited intensity to reduce a patient’s radiation dose. Naïvely training on imperfect images yields an imperfect prior. As a result, another research challenge is accurately learning to model clean images from noisy training images.

Liu et al^93^ and Cui et al^30^ consider Bayesian reconstructions as training data for score learning, to avoid using a point estimate of a noisy image. Considering the whole distribution of possible noisy reconstructions provides additional data from which to infer the clean score function. These self-supervised methods are shown to be competitive with supervised learning approaches, albeit orders of magnitude slower at inference time.

Other approaches by Aali et al^94^^,^^95^ learn an approximate clean score function by introducing further known corruptions to be removed^94^ (an approach best suited to a simple measurement noise model) or by leveraging Stein’s unbiased risk estimate to jointly denoise data and learning its score function.^95^ However, these approaches appear better suited to heavily corrupted image data than the low-level corruption in real medical images.

A different self-supervised approach is taken by Wu et al,^96^ who propose the integration of an adaptive wavelet sub-network in training for denoising and in reconstruction for sparsity regularisation. The wavelet transform is used to preserve image information while reducing noise, but the integration of an additional sparsity prior may interfere with the score-based prior.

### 3D reconstruction

**Figure 11.**
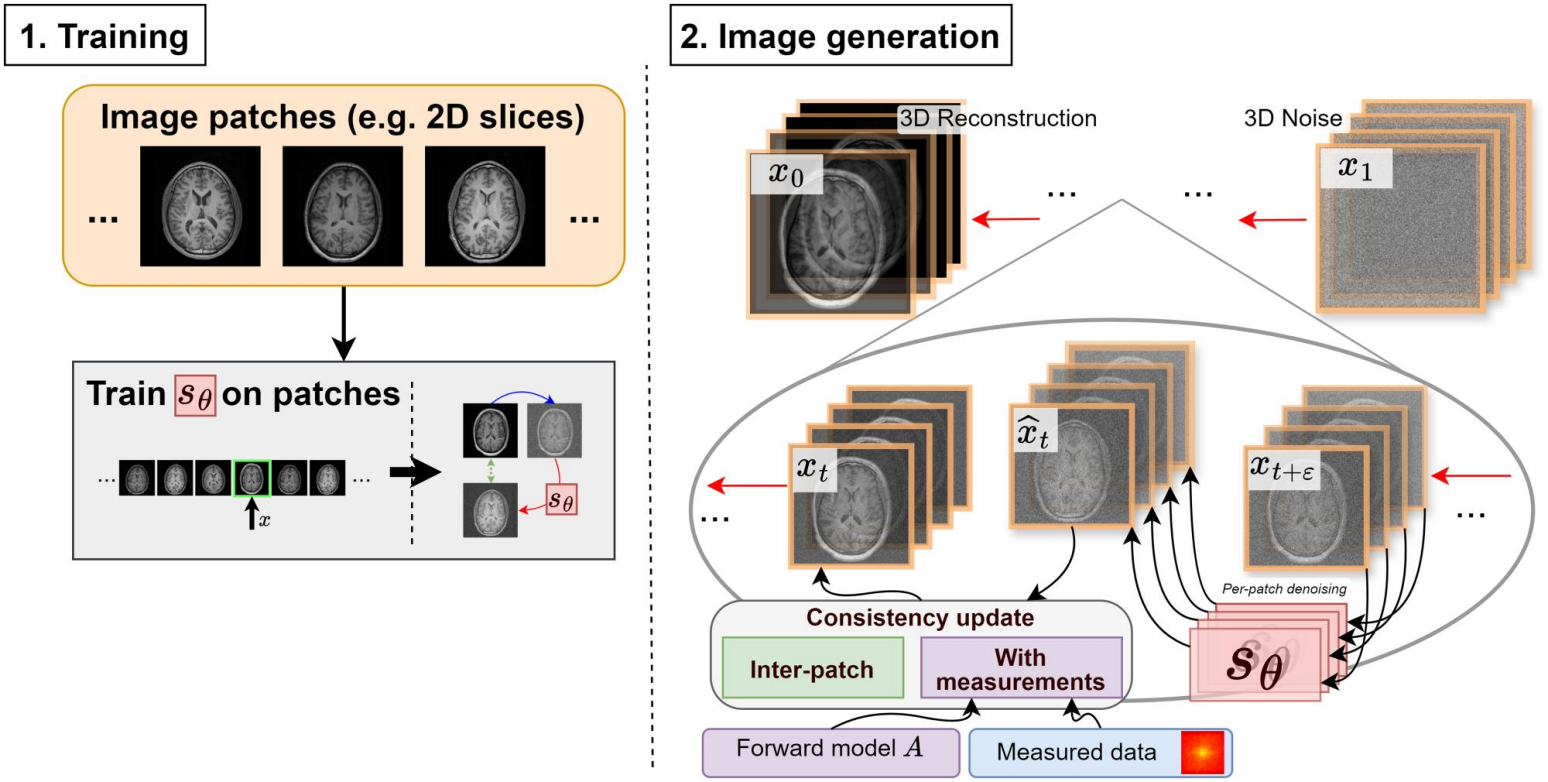
A simple approach to 3D reconstruction with a diffusion model.^97^ A single diffusion model is trained to generate image patches (eg, whole 2D transverse slices). Then, a single diffusion step consists of patch-wise denoising, measurement consistency, and inter-patch consistency updates.

Using a single diffusion model to naïvely generate a full 3D medical volume is computationally infeasible (due to memory limitations). Most authors follow Chung et al^97^ (see Figure 11) by training 1 diffusion model to generate varied 2D parallel slices from a 3D volume, and then reconstructing 2D slices separately with a hand-crafted prior to promoting similarity between neighbouring slices. This is a computationally efficient approach but can lead to slice inconsistency artefacts on slices orthogonal to the diffusion model slice direction.

Lee et al^98^ proposed training multiple diffusion models on perpendicular slice directions, and iterating between the models for reconstruction. This approach does not compromise reconstruction speed while improving slice consistency, only requiring 2–3× memory and training time.

Li et al^99^ instead assume slice similarity across dimensions, proposing evaluating a single trained diffusion model on differently oriented slices to compute a 3D score. This is a strong assumption, and in many cases, it would not be expected to hold in practice. However, computing a 3D score directly rather than iterating between perpendicular scores may reduce the number of iterations required for image generation.

Song et al^20^ instead propose performing the diffusion process in the latent space of a pre-trained variational autoencoder (VAE).^20^^,^^58^ A small latent space could make this approach feasible for 3D reconstruction. However, due to known issues with VAEs such as image compression loss, Chen et al^53^ suggest additional strategies are required to match the fine detail output by pixel-space diffusion models. Furthermore, careful validation of latent models will be required, as encouraging measurement consistency via highly non-linear encoder and decoder functions could yield unexpected effects.

### Alternative diffusion model formulations

Latent diffusion models (see 3D reconstruction) are a promising alternative formulation to pixel-space diffusion models. Latent spaces other than pixel space have the potential for better modelling long-distance dependencies in an image, encouraging emphasis on image details, speeding up image generation, or alleviating memory demands.

As well as learnt latent spaces, pre-defined latent spaces have been proposed, based on the wavelet decomposition (eg, Xu et al^100^) or stacking an image onto itself (eg, Quan et al^25^) which may provide a safer and easier-to-work-with alternative to learnt spaces. In particular, several efforts have focused on the high spatial frequencies corresponding to fine details within an image.^22^^,^^50^^,^^101^

A similarly fine-detail-oriented alternative is learning a patch-based representation of the image (eg, Xia et al^66^) This approach has low memory requirements, allows parallelisation of the image reconstruction process, and can lead to very robust training. However, the focus on textural details within patches may come at the expense of an image’s longer-range spatial dependencies.

Another formulation that various authors^102–104^ have instead proposed is generalised diffusion processes, which replace Gaussian noise with other degradation processes (eg, blurring, spatial frequency removal). Such approaches offer speed increases and may have better alignment with likelihood-based reconstruction processes, but risk losing the superior image quality of standard diffusion models.

Finally, instead of modelling the prior of an image, it is common to model the prior of full acquisition data from limited acquisition data (either solely^24^^,^^54^^,^^62^^,^^105^ or additionally^49^^,^^55^^,^^56^ to the image domain). This approach involves modelling in the measurement domain (k-space for MRI, sinograms for CT or PET), and hence risks losing some of the domain shift resilience of unsupervised diffusion. However, incorporating additional problem-specific information can result in highly successful, albeit less general, approaches.

## Opportunities, challenges, and summary

Diffusion-model-based reconstruction holds the potential to improve image quality in different modalities across different hospital sites and varying scanner configurations. The ability to learn from high-quality images from different scanners (without requiring corresponding measurement data) makes these deep learning models easier to train on clinical data than previous supervised learning methods, raising the eventual possibility of “out-of-the-box” deep learning solutions for reconstruction. Many studies have already shown results for real data demonstrating improvements over the previous state-of-the-art.

Challenges remain for the clinical usage of the algorithms discussed in this article. Researchers are actively engaging with themes including how to avoid hallucinations, accelerate reconstruction times, learn from noisy data, and overcome memory limitations for the reconstruction of large volumes. Few studies have yet been published assessing the clinical impact of images reconstructed by a diffusion model. For breast MRI, Okolie et al^106^ showed at acceleration factor 2 diffusion models produce images almost indistinguishable from the original, as rated by radiologists (for 99% of cases). In the related context of denoising, Xie et al^107^ show significant clinical potential for producing high-quality PET images from low-count data. Integrated reconstruction techniques would be expected to match or surpass post-processing denoising, although similar multi-institutional verification studies will be required to validate this.

In particular, in a clinical setting without ground truth data, tuning the relative strengths of the prior and the likelihood remains a difficult challenge. If the likelihood predominantly guides the reconstruction, in the worst case a noisy or artefact-dominated reconstruction is the output. If instead the diffusion prior predominantly guides the reconstruction, in the worst case a seemingly high-quality reconstruction may be obtained that has hallucinations (image structures or details that are not consistent with reality)—a much more concerning failure mode, as such hallucinations are difficult for clinicians to identify.

Self-supervised techniques to automate the selection of hyperparameters^108^ and tune image generation^87^^,^^88^ could mitigate such concerns.

Researchers can reduce their risk of producing hallucinatory images by robustly verifying their diffusion model has good generalisation properties, by comparing reconstructions to conventional methods, and by assessing images with appropriate quantitative metrics (rather than visually).

Uncertainty quantification may also help alleviate the issue of producing deceptively high-quality but inaccurate reconstructions. By performing multiple reconstructions initialised with different random noise images, we may quantify reconstruction uncertainty at the voxel level (eg, Luo et al^109^) This information has the potential to improve clinical analysis of reconstructed images, although dialogue with clinicians will be essential to establish useful ways to present such information. Uncertainty information could also prove useful to downstream image analysis tasks such as segmentation and classification.

In summary, diffusion models have set new state-of-the-art results for reconstruction tasks across many key medical imaging modalities. The improved robustness to domain shift of unsupervised diffusion models relative to supervised deep learning is a particular strength that will give these models a greater chance of clinical translation. Further work is needed, to speed up reconstruction, reconcile trained priors with measured data, and validate the robustness of reconstructions. Fortunately, the rate of progress in the field is fast, with many avenues remaining for further improvements.
